# Synergistic Charge Storage Enhancement in Supercapacitors via Ti_3_C_2_T_x_ MXene and CoMoO_4_ Nanoparticles

**DOI:** 10.3390/mi15020234

**Published:** 2024-02-01

**Authors:** Christine Young, An-Yi Wu, Ri-Yu Li

**Affiliations:** Functional Nanoporous Materials Laboratory, Department of Chemical and Materials Engineering, National Yunlin University of Science and Technology, Yunlin 640, Taiwan

**Keywords:** MXene, CoMoO_4_, binary transition metal oxides, supercapacitor

## Abstract

MXene has emerged as a highly promising two-dimensional (2D) layered material with inherent advantages as an electrode material, such as a high electrical conductivity and spacious layer distances conducive to efficient ion transport. Despite these merits, the practical implementation faces challenges due to MXene’s low theoretical capacitance and issues related to restacking. In order to overcome these limitations, we undertook a strategic approach by integrating Ti_3_C_2_T_x_ MXene with cobalt molybdate (CoMoO_4_) nanoparticles. The CoMoO_4_ nanoparticles bring to the table rich redox activity, high theoretical capacitance, and exceptional catalytic properties. Employing a facile hydrothermal method, we synthesized CoMoO_4_/Ti_3_C_2_T_x_ heterostructures, leveraging urea as a size-controlling agent for the CoMoO_4_ precursors. This innovative heterostructure design utilizes Ti_3_C_2_T_x_ MXene as a spacer, effectively mitigating excessive agglomeration, while CoMoO_4_ contributes its enhanced redox reaction capabilities. The resulting CoMoO_4_/T_i3_C_2_T_x_ MXene hybrid material exhibited 698 F g^−1^ at a scan rate of 5 mV s^−1^, surpassing that of the individual pristine Ti_3_C_2_T_x_ MXene (1.7 F g^−1^) and CoMoO_4_ materials (501 F g^−1^). This integration presents a promising avenue for optimizing MXene-based electrode materials, addressing challenges and unlocking their full potential in various applications.

## 1. Introduction

With the growing demand for energy storage solutions that are both efficient and sustainable, there is an increasing recognition of the critical significance of electrode materials. The selection of appropriate electrode materials stands as a pivotal factor within the realm of electrochemical energy storage, as these materials exert a direct and profound impact on crucial aspects like the energy density, power density, cycle life, and safety of energy storage devices. By optimizing electrode materials, it becomes possible to propel the performance and sustainability of electrochemical energy storage technologies to new heights.

Commonly used electrode materials encompass carbonaceous materials and a variety of metal oxides and hydroxides. Among these options, transition metal oxides have emerged as highly promising electrode materials for supercapacitors in recent years. Their appeal stems from their remarkable attributes, particularly their notably high theoretical specific capacitance. This enhanced specific capacitance is a consequence of the abundance of redox active sites inherent in these materials, facilitating efficient charge storage and transfer processes. Furthermore, the variable valence states of transition metal ions enable reversible redox reactions to take place at the interface between the electrode and electrolyte, thereby significantly augmenting the overall capacitance of the supercapacitor [1,2,3,4]. In contrast to single-component transition metal oxides, mixed transition metal oxides have gained prominence as compelling electrode materials for supercapacitors. This is primarily attributed to their capacity to provide increased electrical conductivity and a broader spectrum of redox reactions. The amalgamation of various transition metal elements within mixed oxides leads to enhancements in the electrical conductivity, thereby enabling a more efficient charge transfer within the electrode material [5,6,7]. Additionally, the presence of multiple transition metals introduces diverse redox reactions. This allows for a greater variety of charge storage mechanisms to occur, and so, the overall capacitance performance of the supercapacitor can be enhanced. Thus, the exploration of mixed transition metal oxides presents a promising avenue through which the supercapacitor technology can be advanced. Recently, metal molybdates, such as CoMoO_4_, NiMoO_4_, and MnMoO_4_, have demonstrated outstanding electrochemical performances over single component oxides [8,9,10]. In particular, cobalt molybdate (CoMoO_4_) has become highly attractive due to its structural properties and extraordinary capacitance [7]. It is mainly composed of CoO_6_ and MoO_4_ [11,12]. CoMoO_4_ can be prepared into various morphologies based on the synthetic condition. For example, Wang et al. fabricated CoMoO_4_ nanoneeds grown on carbon cloth as a supercapacitor electrode, providing 2100 F g^−1^ at a current density of 1 A g^−1^ [13]. Additionally, Yang et al. prepared a hybrid ZnCo_2_O_4_@CoMoO_4_ heterostructure on Ni foam. It presented the specific capacitance of 1040 C g^−1^ at 1 A g^−1^ [14]. Mei and colleagues effectively produced a nanostructured honeycomb Co–Mo oxide (CoMoO_x_) using a self-assembly approach. The honeycomb-like structure exhibited remarkable structural durability, strong mechanical characteristics, and substantial pore accessibility, resulting in exceptional capabilities for lithium storage [15]. Prasad et al. employed a hydrothermal method and incorporated ethanol into the process to synthesize pebble-like CoMoO_4_ material [16]. The pebble-like CoMoO_4_ exhibited an excellent capacitance retention of 60.4% as the current density was elevated 1.5 to 10 A cm^−2^. Even after undergoing 2000 cycles, the material retained a significant portion of its initial capacitance, specifically 85.95%.

Nevertheless, the utilization of metal oxide nanoparticles as electrode materials comes with a notable challenge: their propensity for agglomeration, which can ultimately diminish their performance. Agglomeration in this context refers to the tendency of metal oxide materials to coalesce into larger clusters or agglomerates during the manufacturing process and their subsequent use as electrodes in electrochemical energy storage devices. This phenomenon arises from various factors, including the elevated surface energy and strong interparticle interactions characterizing metal oxide nanoparticles.

The agglomeration of these nanoparticles introduces substantial hurdles to electrode performance and, by extension, the overall efficiency of the energy storage device. Primarily, agglomerates tend to exhibit reduced surface areas in comparison to individual nanoparticles, leading to a decrease in the number of accessible active sites for electrochemical reactions. This diminishment in the active surface area can have an adverse impact on the electrode’s specific capacity and its energy storage performance. Furthermore, the presence of agglomerates can create diffusion limitations within the electrode’s structure. The larger particle size and the presence of void spaces between these agglomerates impede the efficient transport of ions and electrons, consequently increasing the resistance and slowing down the reaction kinetics. As a result, the power density of the energy storage device may be compromised due to the sluggish charge/discharge rates [17].

Mitigating the challenge of agglomeration in metal oxide materials represents a pivotal research focus within electrode design and fabrication. Diverse strategies are being employed to tackle this issue encompassing surface modifications of nanoparticles, the use of dispersing agents or binders to maintain particle dispersion, and the selection of appropriate solvent systems and processing techniques for controlling particle interactions. By effectively managing and minimizing agglomeration, researchers endeavor to enhance the electrochemical performance, stability, and reliability of electrodes based on metal oxides in the realm of electrochemical energy storage. One strategy to address the agglomeration issue involves the development of hybrid composites through the incorporation of two-dimensional materials renowned for their high conductivity. The primary objective of this integration is to achieve a significant enhancement in the electrochemical performance by capitalizing on the synergistic effects resulting from the distinct energy storage mechanisms exhibited by these materials.

Due to the abundant active sites and substantial surface areas, two-dimensional (2D) materials, such as graphene and zeolite nanosheets, have gained recognition as optimal substrates for catalytic applications [18]. In recent years, a novel category of 2D layered materials, namely, MXenes, has captured significant scientific interest [19]. MXenes are derived from the removal of A layers from M*_n_*_+1_AX*_n_* compounds. Here, M represents a transition metal, A stands for a IIIA or IVA group element, X denotes either C or N, and *n* = 1, 2, 3, or 4. The postetching process yields MXenes, also denoted as Ti_3_C_2_T_x_, with T_x_ signifying surface functional groups such as –OH and –F [20,21,22]. Ti_3_C_2_T_x_ MXenes demonstrate an outstanding conductivity (2.0 × 10^4^ S cm^−1^), possess a two-dimensional structure, feature abundant surface functional groups, and have the capability to enhance electrochemical performance by expanding their interlayer spacing. The unique layered arrangement of Ti_3_C_2_T_x_ MXene provides ample room for anchoring functional nanomaterials, promoting better dispersion on the Ti_3_C_2_T_x_ MXene surface [23,24,25]. This attribute underscores the potential effectiveness of Ti_3_C_2_T_x_ MXenes in addressing the challenge of nanomaterial agglomeration [26]. For example, Tian et al. utilized a flexible and binder-free Si/MXene composite paper as the anode electrode material for lithium-ion batteries [27]. The distinctive structure of this composite paper offers the increased volume expansion space for accommodating stress, and so, this composite paper provided a high capacity of 2118 mAh g^–1^ at a 200 mA g^–1^ current density after 100 cycles, maintaining a steady cycling ability of 1672 mAh g^–1^ at 1000 mA g^–1^ after 200 cycles. Su et al. synthesized ultradispersed binary Fe_3_N/Co–N–C@MXene catalysts for zinc–air battery [28]. Compared to Fe_3_N/Co–N–C, these hybrid materials exhibited uniformly distributed active sites and an enhanced interaction with the electrolyte. The Fe_3_N/Co–N–C@MXene-based zinc–air battery exhibited a higher peak power density (189.16 mW cm^−2^) and stable cycle stability within 320 h.

Based on the aforementioned considerations, this study aimed to fabricate CoMoO_4_/Ti_3_C_2_T_x_ MXene heterogeneous materials. To that end, CoMoO_4_, commonly known for its high theoretical capacitance, was incorporated onto the interlayer and the surface of Ti_3_C_2_T_x_ MXene flakes using a hydrothermal method. Nanosized CoMoO_4_ particles of uniform size were synthesized by using urea as a particle size-controlling agent. The presence of Ti_3_C_2_T_x_ MXene mitigated excessive aggregation during the growth of metal oxides. The comparative analyses demonstrated that the CoMoO_4_/Ti_3_C_2_T_x_ MXene electrode could outperform the pure Ti_3_C_2_T_x_ MXene, single metal oxide/Ti_3_C_2_T_x_ MXene, and pure CoMoO_4_ electrodes in the performances of the supercapacitors.

## 2. Materials

First of all, cobalt nitrate (Co(NO_3_)_2_, 98 wt%), ammonium orthomolybdate ((NH_4_)_2_MoO_4_, 99 wt%), and ammonium fluoride (NH_4_F, 96 wt%) were obtained from Alfa Aesar (Ward Hill, MA, USA). Next, MAX (Ti_3_AlC_2_, ≤40 μm particle size, >90 wt%) and urea (CH_4_N_2_O, >99 wt%) were purchased from Merck (Rahway, NJ, USA). Also, hydrofluoric acid (HF, >48 wt%) was obtained from Honeywell Fluka (Morris Plains, NJ, USA), and polyvinylidene difluoride (PVDF, average MW = 534,000) and *N*-methyl-2-pyrrolidone (NMP, 99 wt%) were purchased from Sigma-Aldrich (St. Louis, MO, USA). Finally, all of these chemicals were used without further purification.

## 3. Synthesis

### 3.1. Synthesis of Ti_3_C_2_T_x_ MXene

A systematic procedure was followed wherein 1 g of MAX was gradually introduced into separate 30 mL solutions of hydrofluoric acid (HF) with concentrations of 48 wt% for 24 h. These solutions were then placed in a temperature-controlled water bath set at a constant temperature of 30 °C. After the completion of the etching process, the precipitates were subjected to multiple rinses with deionized water and underwent centrifugation cycles until the supernatant reached a pH level of approximately 6–7. Following this purification step, the resulting precipitates were collected and subjected to a drying process in an oven maintained at 60 °C overnight.

### 3.2. Synthesis of Needle-like CoMoO_4_

To obtain needle-like CoMoO_4_, the molar ratio of cobalt nitrate to ammonium molybdate was set at 1:1. Ammonium molybdate was dissolved in 70 mL of deionized (DI) water. This was followed by the addition of cobalt nitrate until both were completely dissolved, thereby resulting in a pale pink solution. The pH value of the solution was adjusted to a neutral level using ammonia. It was then stirred for an hour at room temperature and subjected to a hydrothermal reaction at 190 °C for 12 h. After washing and centrifugation, the precipitate was dried at 60 °C overnight and subsequently calcined in a nitrogen atmosphere at 350 °C for 2 h. The resulting product was needle-like CoMoO_4_, designated as *CM*.

### 3.3. Synthesis of Cobalt Oxide/Ti_3_C_2_T_x_ MXene and Molybdenum Oxide/Ti_3_C_2_T_x_ MXene

For cobalt oxide/MXene, the molar ratio of cobalt nitrate to Ti_3_C_2_T_x_ MXene was 1:1. Cobalt nitrate was dissolved in 70 mL of water, followed by Ti_3_C_2_T_x_ MXene addition. The mixture was then transferred to a reaction vessel and subjected to hydrothermal treatment at 150 °C for 12 h within an oven. After that, the product was washed and centrifuged multiple times using DI water. The resulting precipitate was dried at 60 °C overnight and subsequently calcined in a nitrogen atmosphere at 350 °C for 2 h. This process yielded cobalt oxide/Ti_3_C_2_T_x_ MXene, labeled as *CoX*.

Similarly, for the fabrication of molybdenum oxide/Ti_3_C_2_T_x_ MXene, the molar ratio of ammonium molybdate to Ti_3_C_2_T_x_ MXene was also set at 1:1. The production process was analogous to the aforementioned procedure. The resulting product was designated as *MoX*.

### 3.4. Synthesis of CoMoO_4_/Ti_3_C_2_T_x_ MXene

In this fabrication process, a molar ratio of 1:1:1:2.5:1 for Ti_3_C_2_T_x_ MXene, cobalt nitrate, ammonium molybdate, urea, and ammonium fluoride was considered. Initially, ammonium molybdate was dissolved in 70 mL of DI water. Following this, cobalt nitrate was completely dissolved in the aforementioned solution. Subsequently, a mixture of urea and ammonium fluoride was introduced and stirred for 10 min. This was followed by the addition of Ti_3_C_2_T_x_ MXene. After being stirred for an hour at room temperature, the mixture was transferred to an autoclave and subjected to hydrothermal treatment at 150 °C for 12 h in an oven. The resulting precipitate was collected and washed multiple times. Afterwards, the resulting precipitate was dried at 60 °C overnight and then subjected to calcination in a nitrogen atmosphere at 350 °C for 2 h. This process yielded CoMoO_4_/Ti_3_C_2_T_x_ MXene, designated as *CMX-1*.

To explore the impact of the urea content on the dispersion of cobalt molybdate within Ti_3_C_2_T_x_ MXene, two additional samples were synthesized with varying urea ratios. One had a molar ratio of 1:1:1:1:1 for cobalt nitrate, ammonium molybdate, urea, ammonium fluoride, and Ti_3_C_2_T_x_ MXene, marked as *CMX-2*. The other had a molar ratio of 1:1:1:5:1 for the same components, labeled as *CMX-3*.

## 4. Characterization

The morphologies of the prepared samples were examined through two different techniques: scanning electron microscopy (SEM), using a JEOL JSM-6701F instrument (JEOL, Tokyo, Japan) and transmission electron microscopy (TEM), employing a JEOL JEM-2100Plus microscope (JEOL, Tokyo, Japan). For the X-ray diffraction pattern analysis, an X-ray diffractometry (XRD) instrument, specifically the MiniFlex-600 from Rigaku (Tokyo, Japan), was utilized, equipped with Cu Kα radiation. Raman spectra were acquired using an Invia Raman microscope by Renishaw (Wharton Anderch, UK), featuring a 633 nm laser source. To conduct nitrogen adsorption–desorption measurements, an autoSorb iQ-TPX instrument by Quantachrome (New York, NY, USA) was employed. The specific surface area was determined using the Brunauer–Emmett–Teller (BET) theory, and the pore size distribution was determined using the Barrett–Joyner–Halenda (BJH) method. The chemical state analysis was performed using X-ray photoelectron spectroscopy (XPS) with a PHI Versa Probe 4 instrument (Physical Electronics, Chanhassen, MN, USA), employing Al Kα radiation.

### Electrochemical Measurements

All electrochemical tests were carried out using an SP-150e instrument from BioLogic (Seyssinet-Pariset, France). The electrode fabrication process involved the combination of active materials, super P as a conductive additive and polyvinylidene fluoride (PVDF) as a binder, in an 8:1:1 ratio. Subsequently, 200 μL of NMP (*N*-methyl-2-pyrrolidone) was added as a solvent. The components were thoroughly mixed using an ultrasonic oscillator until they were well dispersed. The resulting mixture was then incrementally and evenly applied onto a nickel foam substrate. The assembled electrode was placed in a vacuum oven at 60 °C for overnight drying to ensure complete evaporation of the NMP solvent. For the three-electrode system, the working electrodes were prepared using various active material samples, including Ti_3_C_2_T_x_ MXene, CM-1, CM-2, CoX, MoX, CMX-1, CMX-2, and CMX-3. In the electrochemical setup, Pt foil served as the counter electrode, while a Hg/HgO electrode was employed as the reference electrode. The electrolyte used for these experiments was a 6 M KOH solution. Three-electrode tests were employed: cyclic voltammetry (CV) with scan rates set at 5, 10, 20, 40, 60, 80, and 100 mV s^–1^; galvanostatic charge–discharge (GCD) tests with current densities of 0.5, 1, 2, 5, and 10 A g^–1^; and electrochemical impedance spectroscopy (EIS) over a frequency range from 10^–3^ Hz to 1 MHz. The specific capacitance was calculated based on the following equation:

(1) $$C=\frac{\int idV}{2mvV}$$

where “*i*” is the applied current (A), “*V*” is the potential range (V), “*m*” is the mass of the active material (g), and “*v*” is the scan rate (V s^–1^).

## 5. Results and Discussion

**Figure 1:**
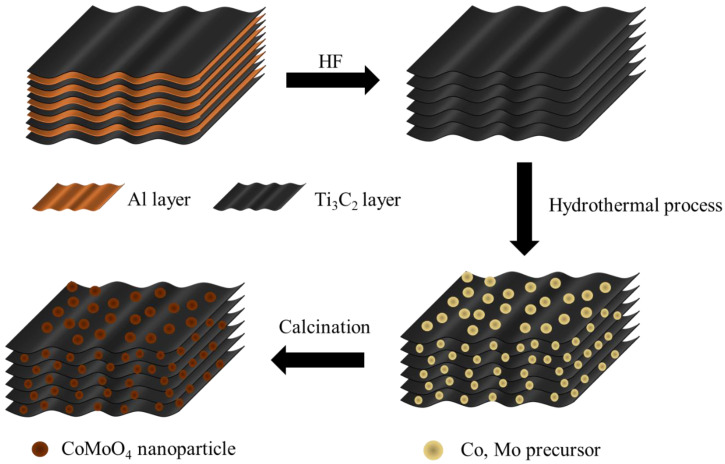
Schematic illustration of the fabrication process for CMX heterostructure (CoMoO_4_/Ti_3_C_2_T_x_ MXene) samples.

The synthesis of the CMX samples was a straightforward yet effective hydrothermal process in which a meticulous calcination step was involved, as illustrated in Figure 1. Initially, MAX was subjected to HF etching to eliminate the atomic aluminum layer, thereby yielding pristine Ti_3_C_2_T_x_ MXene. Subsequently, a hydrothermal reaction was orchestrated to foster the growth of Co and Mo precursor nanoparticles directly onto the Ti_3_C_2_T_x_ MXene substrate. Finally, the carefully controlled calcination process was employed to transform these precursors into the coveted CoMoO_4_ phase. These meticulously executed steps resulted in the formation of CoMoO_4_/Ti_3_C_2_T_x_ MXene composites with a sophisticated three-dimensional heterostructure.

**Figure 2:**
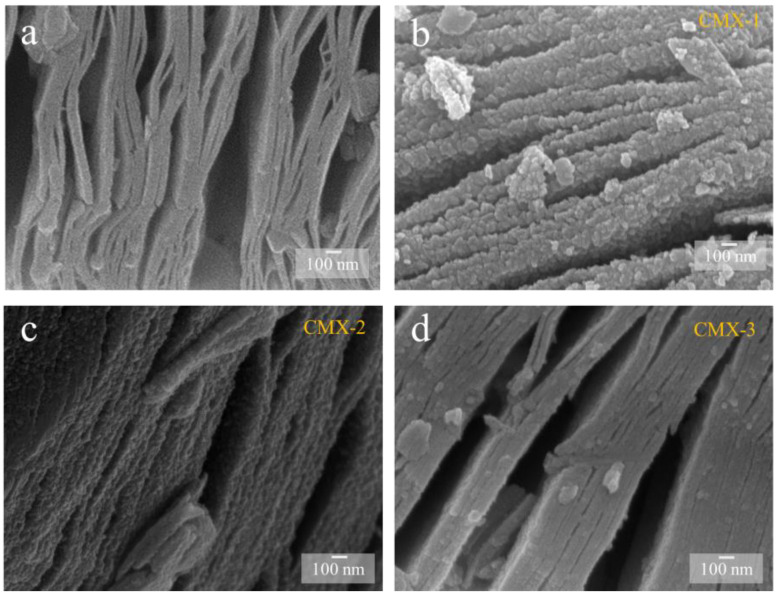
SEM images of (**a**) Ti_3_C_2_T_x_ MXene, (**b**) CMX-1, (**c**) CMX-2, and (**d**) CMX-3 samples.

In order to better obtain Ti_3_C_2_T_x_ MXene, MAX is usually required to undergo an etching process. Figure S1a shows the MAX material. This material displays a typical layered structure in which the layers are tightly interconnected to form a dense morphology. When etched with 48 wt% HF, the resulting Ti_3_C_2_T_x_ MXene sample (Figure 2a) exhibited evident layers of structures with a characteristic accordion-like morphology. This suggests a highly successful etching process for the production of MXene. The results generated from the analysis of the XRD crystal structure of MAX and Ti_3_C_2_T_x_ MXene are provided in Figure S1b; the measurement was in the 2θ ranges of 5° to 70°. The MAX sample exhibited characteristic peaks of MAX materials at 2θ values of 9.4°, 19.1°, 33.9°, 36.7°, 38.7°, 41.6°, 48.4°, 56.2°, and 60.1°, corresponding to the (002), (004), (101), (103), (104), (105), (107), (108), and (110) crystal planes’ diffraction peaks [29]. In the Ti_3_C_2_T_x_ MXene sample, at 2θ values of 8.8°, 18.5°, 27.8°, 34.1°, 41.7°, and 60.5°, peaks corresponding to the (002), (004), (006), (008), (0012), and (110) crystal planes were observed. This part of the finding is consistent with that which is documented in the referenced literature related to Ti_3_C_2_T_x_ MXene [30]. Notably, the (104) crystal plane was absent in the Ti_3_C_2_T_x_ MXene sample, which indicates the removal of the aluminum layer. As shown in Figure S1c, it can be observed that the (002) crystal plane peak of Ti_3_C_2_T_x_ MXene shifts leftward, from 2θ = 9.4° to 8.8°. This is because that the interlayer spacing has increased after the etching process. The Raman spectrum of Ti_3_C_2_T_x_ MXene can possibly exhibit multiple characteristic peaks in the range of 100–800 cm^−1^ [31]. A wavelength of 207 cm^−1^ arises from the A_1g_ (Ti, C, and T_x_) composition within the laminar regions. This wavelength is associated with the in-plane and out-of-plane vibrations of the outermost Ti atoms, as well as the vibrations of carbon and surface terminations [32]. The spectral range spanning from 250 to 470 cm^−1^ corresponds to in-plane (E_g_) vibrations of surface terminations connected to Ti atoms. In the 580–730 cm^−1^ range, the vibrations are primarily attributed to carbon-related vibrations. It is apparent that these vibrations are limited due to the expansion and stacking of interlayer spacing. The introduction of intercalants, which leads to an increase in the water content between layers, enhances out-of-plane vibrations. A larger interlayer spacing results in a shift of the A_1g_ (C) peak from approximately 712 to 722 cm^−1^.

**Figure 3:**
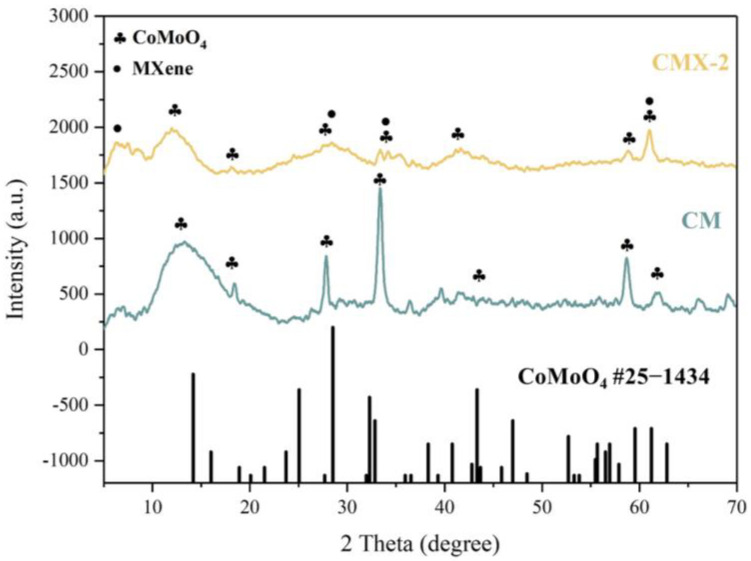
XRD patterns of CM and CMX-2 samples.

**Figure 4:**
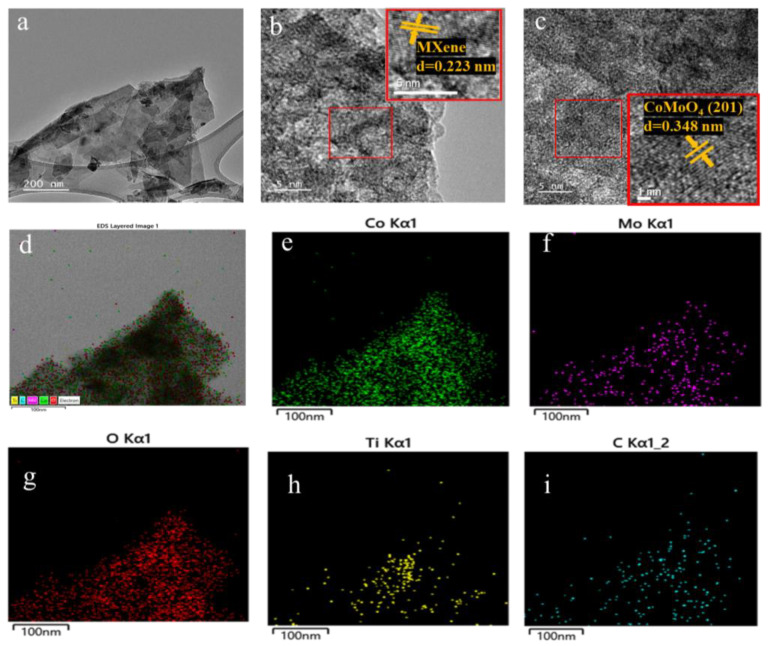
(**a**) TEM and (**b**,**c**) HRTEM images of CMX-2 samples. (**d**–**i**) EDX mapping of CMX-2 samples.

In order to obtain the CoMoO_4_/Ti_3_C_2_T_x_ MXene heterostructure, different concentrations of urea were employed to regulate the crystal formation and growth of CoMoO_4_. T This process yielded three distinct samples with varying urea ratios, designated as CMX-1, CMX-2, and CMX-3, as illustrated in Figure 2b–d. It is important to note that urea is commonly used as a precipitating agent in the coprecipitation method due to its high solubility in water and its ability to control the rate of hydrolysis, leading to the production of ammonium and hydroxide ions through temperature control [33]. Urea gradually generates hydroxyl groups, and so, this results in a slow precipitation process that facilitates the formation of uniformly dispersed particles, thereby reducing aggregation. Within these samples, CMX-1 featured substantial and uniform CoMoO_4_ crystal particles distributed on the Ti_3_C_2_T_x_ MXene surface. CMX-2, which had the lowest urea ratio, exhibited consistently small-sized and evenly distributed CoMoO_4_ particles that grew between the Ti_3_C_2_T_x_ MXene layers. In contrast, CMX-3, with the highest urea ratio, showed fewer CoMoO_4_ particles, indicating the insufficient growth of CoMoO_4_ nanoparticles. This observation underscores the fact that modifying the urea concentration does, in fact, influence the growth characteristics of CoMoO_4_ crystals. In Figure 3 and Figure S3a, the XRD analysis of CMX-1, CMX-2, and CMX-3 exhibited distinct diffraction peaks at 2θ values of 14.1°, 18.9°, 28.5°, 32.3°, 43.3°, 59.5°, and 61.2°. These correspond to the crystal planes (110), (−201), (220), (−222), (−330), (−351), and (152) of CoMoO_4_ (JCPDS #25-1434), respectively. Additionally, diffraction peaks corresponding to Ti_3_C_2_T_x_ MXene were also observed. TEM and HRTEM images of CMX-2 are presented in Figure 4a–c, revealing the well-crystallized structure of these hybrid materials. The lattice spacings of 0.223 and 0.348 nm can be ascribed to (002) plane of Ti_3_C_2_T_x_ MXene and (−201) plane of CoMoO_4_, respectively. Furthermore, the EDS elemental mapping results, as displayed in Figure 4d–i, confirmed the presence of Co, Mo, O, C, and Ti elements in the CMX-2 hybrid materials.

For comparison, the Co- and Mo-based precursor undergoing the hydrothermal and calcination process was adopted to prepare CoMoO_4_ without Ti_3_C_2_T_x_ MXene. As clearly shown in Figure S2a, the CM sample showed a needle-like structure. To compare with the single element transition metal oxide on Ti_3_C_2_T_x_ MXene, Co- and Mo-based precursors were integrated with Ti_3_C_2_T_x_ MXene, respectively, which were denoted as CoX and MoX, as can be seen in Figure S2b,c. The CoX sample exhibited noticeable particles. This suggests that the particle size of the synthesized Co_3_O_4_ could be larger than the interlayer spacing of Ti_3_C_2_T_x_ MXene. As a result, Co_3_O_4_ tends to struggle to effectively grow between the layers of Ti_3_C_2_T_x_ MXene, thereby leading to a significant dispersion of metal oxide particles on the exterior of the Ti_3_C_2_T_x_ MXene layers. In Figure S2c, no apparent particle formations were observed. This indicates that only a small amount of MoO_3_ could grow onto the Ti_3_C_2_T_x_ MXene. CoX and MoX revealed distinct diffraction peaks, corresponding to the crystal planes of CoMoO_4_ (JCPDS #25-1434), Co_3_O_4_ (JCPDS #42-1467), and MoO_3_ (JCPDS #05-0508), respectively. Among all the composites, the diffraction peaks of residual Ti_3_C_2_T_x_ MXene were observed. Therefore, the XRD results suggest that the CMX-1, CMX-2, CMX-3, CoX, and MoX composites could be successfully synthesized.

**Figure 5:**
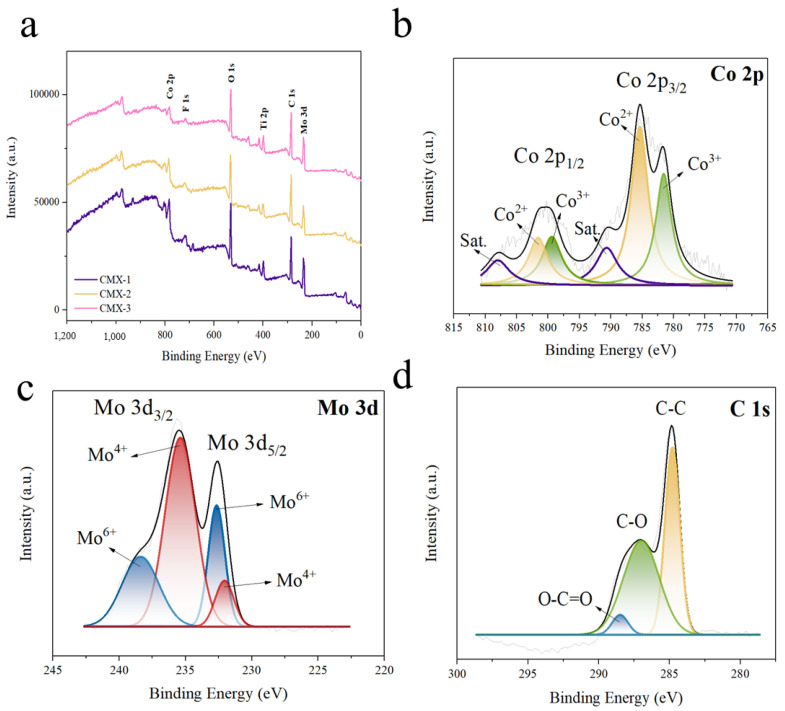
(**a**) Full XPS spectrum and high-resolution XPS spectra with raw data and fitted curves for the (**b**) Co 2p, (**c**) Mo 3d, and (**d**) C 1s peaks in CMX-2.

The technique of XPS analysis was executed to investigate the valence states of CMX-1, CMX-2, and CMX-3. The full XPS spectra are depicted in Figure 5, illustrating the presence of Co, Mo, O, C, and Ti elements in all samples. The element component ratio can be found in Table S1. Notably, all samples exhibited a substantial carbon content and included Ti elements, signifying the prominent existence of the Ti_3_C_2_T_x_ MXene material. Additionally, the aluminum content in all three samples was observed to be below 0.1 at%, indicating the successful removal of the aluminum layer from the MAX phase. The high-resolution XPS spectra of CMX-1, CMX-2, and CMX-3 are individually presented in Figure 5, Figures S4 and S5, respectively. In the Co 2p spectrum, the peaks were fitted into satellites and two spin–orbit doublets. The peaks at 785.1 eV and 801.5 eV were attributed to Co^2+^, while those at 781.8 eV and 799.5 eV corresponded to Co^3+^. This spectral analysis corroborates the presence of cobalt oxide [34]. In the Mo 3d spectrum, the peaks were deconvoluted into satellites and two spin–orbit doublets, Mo 3d_3/2_ and Mo 3d_5/2_. The peaks at 232.2 and 235.5 eV were assigned to Mo^4+^, while the peaks at 232.8 and 238.4 eV were ascribed to Mo^6+^ [35]. In the C 1s spectrum, the peaks at 284.8 eV, 287.1 eV, and 288.5 eV corresponded to C–C bonds, C–O bonds, and O–C=O bonds, respectively [36]. The amalgamation of the XRD, TEM, and XPS results provides compelling evidence that the material consists of a combination of CoMoO_4_ and Ti_3_C_2_T_x_ MXene.

The porous structures of Ti_3_C_2_T_x_ MXene and CMX-2 were investigated using nitrogen adsorption–desorption isotherms, as shown in Figure S6. Ti_3_C_2_T_x_ MXene showed a specific surface area of 2.2 m^2^ g^−1^ and an average pore size of 0.097 nm, while CMX-2, in contrast, showed a much larger specific surface area of 38.9 m^2^ g^−1^ and an average pore size of 0.150 nm. This indicates that the introduction of CoMoO_4_ between the Ti_3_C_2_T_x_ MXene layers could significantly enlarge the surface area, which may facilitate the transportation of ions in the electrolyte for electrochemical applications.

**Figure 6:**
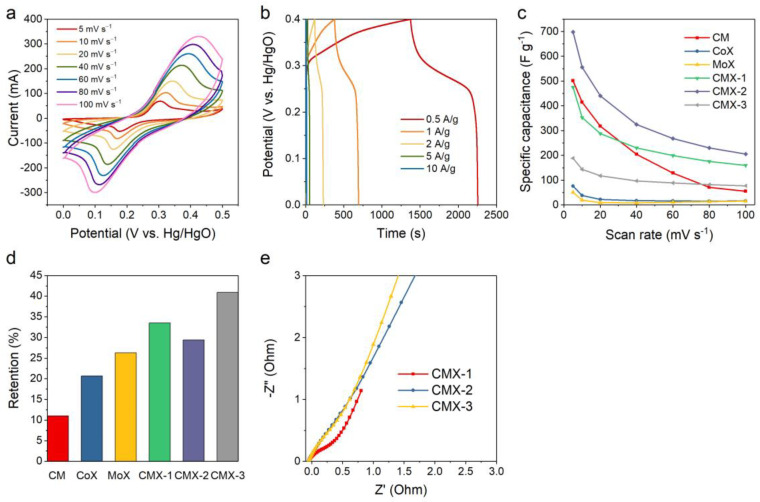
(**a**) CV curves and (**b**) GCD curves of CMX-2. (**c**,**d**) Capacitances and capacitance retentions for CM, CoX, MoX, CMX-2, CMX-2, and CMX-3 at various scan rates. (**e**) Nyquist plots for CMX-1, CMX-2, and CMX-3.

The electrochemical performances of CM, CoX, MoX, CMX-1, CMX-2, and CMX-3 were analyzed using a three-electrode system in a 6 M KOH aqueous electrolyte. The results from cyclic voltammetry (CV) and galvanostatic charge−discharge (GCD) measurement of CMX-2 and other samples are shown in Figure 6, Figures S7 and S8. The CV curves showed high redox peak currents and large enclosed areas which reveal typical pseudocapacitive characteristics. The optimal potential window was adjusted to 0−0.5 V. The specific capacitances of Ti_3_C_2_T_x_ MXene, CM, CoX, MoX, CMX-1, CMX-2, and CMX-3 at a scan rate of 5 mV s^−1^ were calculated to be 1.7, 501, 76, 51, 475, 698, and 189 F g^−1^, respectively. Figure 6c shows the specific capacitances of all the samples at various scan rates of 5 to 100 mV s^−1^, and the corresponding retentions for all the samples were 45.7, 11.0, 20.7, 26.3, 33.5, 29.4, and 40.9%, respectively, as presented in Figure 6d. CMX-2 possessed the highest specific capacitance among the samples. Compared to CMX-1 and CMX-3, it had a well-defined layered structure. This structure can hinder the aggregation of crystals and so enhance the structural stability. In addition, the uniformity of CoMoO_4_ nanoparticle growth on the Ti_3_C_2_T_x_ MXene layer rendered a sufficient Faradaic reaction, which may in turn facilitate the transportation and diffusion of electrolyte ions. Ti_3_C_2_T_x_ MXene had nearly no capacitance contribution at this range of operation voltage window (Figure S7a,b). The CM sample had a relatively high specific capacitance owing to the needle-like morphology. However, it is evident that without the Ti_3_C_2_T_x_ MXene layered structure, it suffered from an aggregation problem. This led to the lowest capacitance retention (11.0%). Due to their integration with Ti_3_C_2_T_x_ MXene, CoX and MoX exhibited enhanced capacitance retentions compared to the CM sample. Nevertheless, their relatively low specific capacitances can be attributed to the insufficient occurrence of Faradaic reactions within a single-metal system [37]. The EIS measurements were conducted to evaluate the impedance of CMX-1, CMX-2, and CMX-3. As shown in Figure 6e, the Nyquist plots unveiled a notable semicircular feature in the high-frequency range, followed by a linear portion in the low-frequency region. Notably, in the case of CMX-1, the semicircle exhibited a larger diameter, and the straight line had a lower slope. This observation implies a relatively higher level of ionic diffusion resistance and charge transfer resistance. This phenomenon can be predominantly attributed to the aggregation of CoMoO_4_ on the Ti_3_C_2_T_x_ MXene layer. It is evident that the performance of CMX-2 and CMX-3 is quite similar. Despite CMX-3 demonstrating comparable electrical conductivity, the absence of observable CoMoO_4_ nanoparticles on the Ti_3_C_2_T_x_ MXene surface resulted in a poor capacitive performance. Comparing the impedances of the Ti_3_C_2_T_x_ MXene, CM, MoX, and CMX-2 samples (Figure S9) makes it evident that Ti_3_C_2_T_x_ Mxene exhibited the highest electrical conductivity, followed by CMX-2, MoX, and CM. CM, lacking the addition of Ti_3_C_2_T_x_ Mxene, exhibited the poorest conductivity, which subsequently led to the lowest capacitive retention performance, as discussed earlier. CMX-2, benefiting from its binary metal composition, exhibited a better conductivity than MoX. Consequently, these results strongly underscore the advantages of the integrated structure of Ti_3_C_2_T_x_ MXene and CoMoO_4_.

To investigate the electrochemical kinetics involved in charge storage for the CMX-1, CMX-2, and CMX-3 electrode materials, cyclic voltammetry (CV) measurements were conducted over a range of scan rates, spanning from 0.1 to 2 mV s^−1^. This analysis aimed to delve into the intricate dynamics of charge storage mechanisms, which encompass both diffusion-controlled and surface-controlled kinetics. Typically, the electrode’s current exhibits a power law relationship between the current density and the scan rate, as represented by the following equation [38]:

*i* = *av^b^* (2)

**Figure 7:**
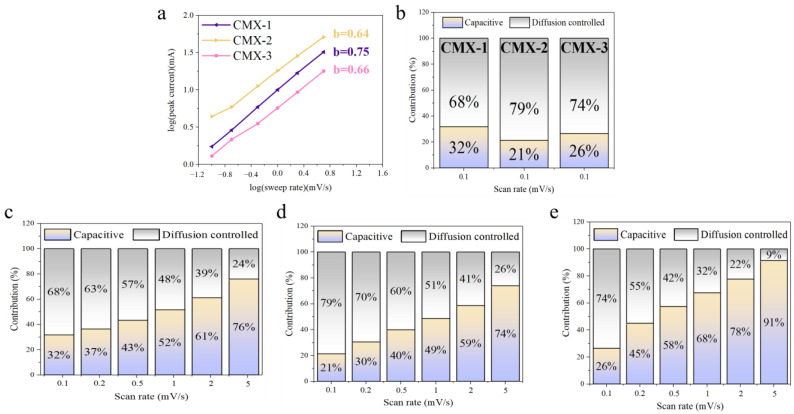
(**a**) The relation curve of redox peaks to scan rates of CMX-1, CMX-2, and CMX-3. (**b**) Evaluation of the surface capacitive and diffusion-controlled charge storage mechanisms at the scan rate of 0.1 mV s^−1^. Capacitive and diffusion-controlled contribution at various scan rates for (**c**) CMX-1, (**d**) CMX-2, and (**e**) CMX-3.

The *b*-value is the slope of the plot of log *i* vs. log *v* [39]. A *b*-value of 0.5 is a common indicator of a diffusion-controlled process, while a *b*-value of 1 indicates a capacitive-controlled process. As depicted at Figure 7a, the *b*-values CMX-1, CMX-2, and CMX-3 were 0.75, 0.64, and 0.66, respectively. Significantly, it is important to highlight that CMX-2 exhibits a *b*-value that closely approximates 0.5, indicating a diffusion-controlled behavior that significantly contributes to facilitating rapid redox reaction kinetics and ion diffusion throughout the process. This suggests that CMX-2 could offer an enhanced performance due to its advantageous charge storage mechanism primarily driven by diffusion control. Moreover, the quantitative comparisons between high-speed and slow kinetic processes can be made using the two equations provided below [40]:

(3) $$i\left(V\right)=k_{1}v+k_{2}v^{\frac{1}{2}}$$

The parameter “*i*” represents the current at voltage V, while “*k*_1_*v*”and “*k*_2_*v*^1/2^” correspond to the capacitive and diffusion-controlled process. The *k*_1_-value and *k*_2_-value can be determined from the linear relationship of the *k*_1_*v* and *k*_2_*v*^1/2^. Figure 7b–d and Figures S10–S12 illustrate the percentages of capacitive and diffusion-controlled processes of CMX-1, CMX-2, and CMX-3. At a scan rate of 0.1 mV s^−1^, CMX-1, CMX-2, and CMX-3 samples exhibited 68%, 79%, and 74% in diffusion-controlled processes. Notably, at all scan rates, the CMX-2 electrode showed a higher proportion of diffusion-controlled contribution compared to the other two electrodes. This could be attributed to the well-defined layered structure of MXene/CoMoO_4_ and uniformly distributed nanoparticles of CoMoO_4_ in CMX-2, which facilitates the easier permeation of OH^−^ ions. The diffusion-controlled capacitances of the CMX-2 electrode were found to be 79%, 70%, 60%, 51%, 41%, and 26% at scan rates of 0.1, 0.2, 0.4, 0.6, 0.8, and 1 mV s^−1^, respectively. This suggests that at low scan rates, the diffusion process predominantly governs, while at higher rates, the surface capacitive process takes control due to a limited diffusion time. Moreover, the significant predominance of the diffusion-controlled contribution to the overall capacitance implies a relatively constrained rate capability for the CMX-2 electrode, consistent with the earlier electrochemical results. The cycling stabilities were conducted in the scan rate of 40 mV s^−1^ for 1000 cycles. The decreases in capacitance are 83.4%, 82.8%, and 83.8% for the CMX-1, CMX2, and CMX-3 electrode. After the cycling test, the CMX-2 electrode material underwent SEM analysis. As depicted in Figure S13a,b, the Ti_3_C_2_T_x_ MXene layer retained its characteristic layered structure. However, the CoMoO_4_ particles exhibited a tendency to agglomerate into larger clusters. This aggregation is likely to result in accumulated stress, ultimately leading to degradation during the cyclic stability test. Therefore, enhancing the stability of the MXene/CoMoO_4_ heterostructure as a supercapacitor device electrode in the future may hinge on achieving a more robust morphology for CoMoO_4_ nanoparticles.

In summary, we have successfully integrated Ti_3_C_2_T_x_ MXene and CoMoO_4_, resulting in the formation of the CoMoO_4_/Ti_3_C_2_T_x_ MXene heterostructure with a distinctive three-dimensional layered morphology. The enhanced electrochemical performance of the CoMoO_4_/Ti_3_C_2_T_x_ MXene heterostructure (698 F g^−1^ at a scan rate of 5 mV s^−1^) can be attributed to several factors. First, Ti_3_C_2_T_x_ MXene plays a crucial role in mitigating aggregation, thereby facilitating the electrochemical reactions. Additionally, the anchoring of CoMoO_4_ nanoparticles onto Ti_3_C_2_T_x_ MXene leads to an increased surface area, providing abundant electrochemical active sites. The remarkable specific capacitance of CMX-2 can be attributed to the uniform distribution of CoMoO_4_ nanoparticles and the stable three-dimensional hollow structure it possesses. These findings underscore the importance of manipulating metal oxide nanoparticle morphologies within the Ti_3_C_2_T_x_ MXene structure. This becomes particularly significant in the context of advancing innovative electrode materials for high-performance supercapacitors. The successful integration of Ti_3_C_2_T_x_ MXene and CoMoO_4_ and the resulting improvements in the electrochemical performance hold promise for the development of next-generation energy storage devices.

## 6. Conclusions

In summary, our research has successfully demonstrated a straightforward method through which CoMoO_4_/Ti_3_C_2_T_x_ MXene heterostructures can be synthesized. Also, this method can set them apart from both individual MXene/single metal oxide composites (CoX and MoX) and standalone CoMoO_4_ materials (CM). In this investigation, we utilized urea to yield well-dispersed cobalt molybdate nanoparticles, and they were strategically deposited onto the surface and within the interlayer spaces of the Ti_3_C_2_T_x_ MXene matrix. This innovative approach was validated, because it effectively mitigated the issue of excessive aggregation often encountered during the growth of metal oxide materials. CMX-1, CMX-2, and CMX-3 exhibited the specific capacitances of 475, 697.5, and 189 F g ^–1^ at a scan rate of 5 mV s^–1^, respectively. In contrast, CoX and MoX exhibited substantially lower specific capacitances, thereby underscoring the inferior electrochemical performance of individual metal oxides. Meanwhile, CM, due to a lack of the Ti_3_C_2_T_x_ MXene component, not only displayed a lower specific capacitance, but also suffered from diminished capacitance retention at high scan rates. This indicates a deficiency in the ion transport. Additionally, this remarkable electrochemical performance can be attributed to a combination of factors: firstly, the well-constructed CoMoO_4_/Ti_3_C_2_T_x_ MXene heterostructure, which enhances the accessibility to electrochemically active sites; secondly, the lower resistance inherent in this composite architecture compared to other samples; and thirdly, the favorable faradic reactions facilitated by CoMoO_4_. Consequently, we strongly recommend further investigation into this approach for the fabrication of metal oxides/MXene heterostructures with enhanced electrochemical activities, so as to further offer promising prospects for future research in this domain.

## Data Availability

The data presented in this study are available on request from the corresponding author.

## Supplementary Materials

The following supporting information can be downloaded at: https://www.mdpi.com/article/10.3390/mi15020234/s1, Figure S1. (a) SEM image of MAX. (b,c) XRD patterns of MAX and Ti_3_C_2_T_x_ MXene. (d) Raman spectrum of Ti_3_C_2_T_x_ MXene. Figure 2. SEM images of (a) CM, (b) CoX, and (c) MoX. Figure S3. (a) XRD patterns of CMX-1, CMX-2, and CMX-3. (b) XRD patterns of Ti_3_C_2_T_x_ MXene, CoX, and MoX. Figure S4. High resolution XPS spectra showing the (a) Co 2p, (b) Mo 3d and (c) C 1s for CMX-1. Figure S5. High resolution XPS spectra showing the (a) Co 2p, (b) Mo 3d and (c) C 1s for CMX-3. Figure S6. (a) Nitrogen adsorption-desorption isotherms for Ti_3_C_2_T_x_ MXene and CMX-2. (b) Pore size distribution of Ti_3_C_2_T_x_ MXene and CMX-2 determined using BJH method. Figure S7. CV curves and GCD curves of (a,b) Ti_3_C_2_T_x_ MXene, (c,d) CM, (e,f) CoX, and (g,h) MoX. Figure S8. CV curves and GCD curves of (a,b) CMX-1, (c,d) CMX-2, and (e,f) CMX-3. Figure S9. The Nyquist plot of Ti_3_C_2_T_x_ MXene, CM, MoX, and CMX-2. Figure S10. (a) CV curves of CMX-1 at the scan rates of 0.1, 0.2, 0.5, 1, 2, 5 mV s^−1^. (b) Capacitive and diffusion controlled contribution at various scan rates for CMX-1. (c–h) CV curves showing the capacitive and diffusion-controlled contributions at the scan rates ranging from 0.1 to 5 mV s^−1^. Figure S11. (a) CV curves of CMX-2 at the scan rates of 0.1, 0.2, 0.5, 1, 2, 5 mV s^−1^. (b) Capacitive and diffusion controlled contribution at various scan rates for CMX-2. (c–h) CV curves showing the capacitive and diffusion-controlled contributions at the scan rates ranging from 0.1 to 5 mV s^−1^. Figure S12. (a) CV curves of CMX-3 at the scan rates of 0.1, 0.2, 0.5, 1, 2, 5 mV s^−1^. (b) Capacitive and diffusion controlled contribution at various scan rates for CMX-3. (c–h) CV curves showing the capacitive and diffusion-controlled contributions at the scan rates ranging from 0.1 to 5 mV s^−1^. Figure S13. SEM images of CMX-2 after stability test. Table S1. The element component ratio of CMX-1, CMX-2, and CMX-3.
