# Supplementary material for: Synergistic Charge Storage Enhancement in Supercapacitors via Ti3C2Tx MXene and CoMoO4 Nanoparticles
Source: Micromachines (Basel). 2024 Feb 1;15(2):234. doi: 10.3390/mi15020234 (PMC10893255; doi:10.3390/mi15020234)
Supplement: Supplementary file 1 [file micromachines-15-00234-s001.zip › micromachines-2842051-supplementary.pdf]

# Supplementary Material

# Synergistic Charge Storage Enhancement in Supercapacitors via $Ti_3C_2T_x$ MXene and $CoMoO_4$ Nanoparticles

Christine Young *, An-Yi Wu and Ri-Yu Li

*Functional Nanoporous Materials Laboratory, Department of Chemical and Materials Engineering, National Yunlin University of Science and Technology, Yunlin 640, Taiwan*

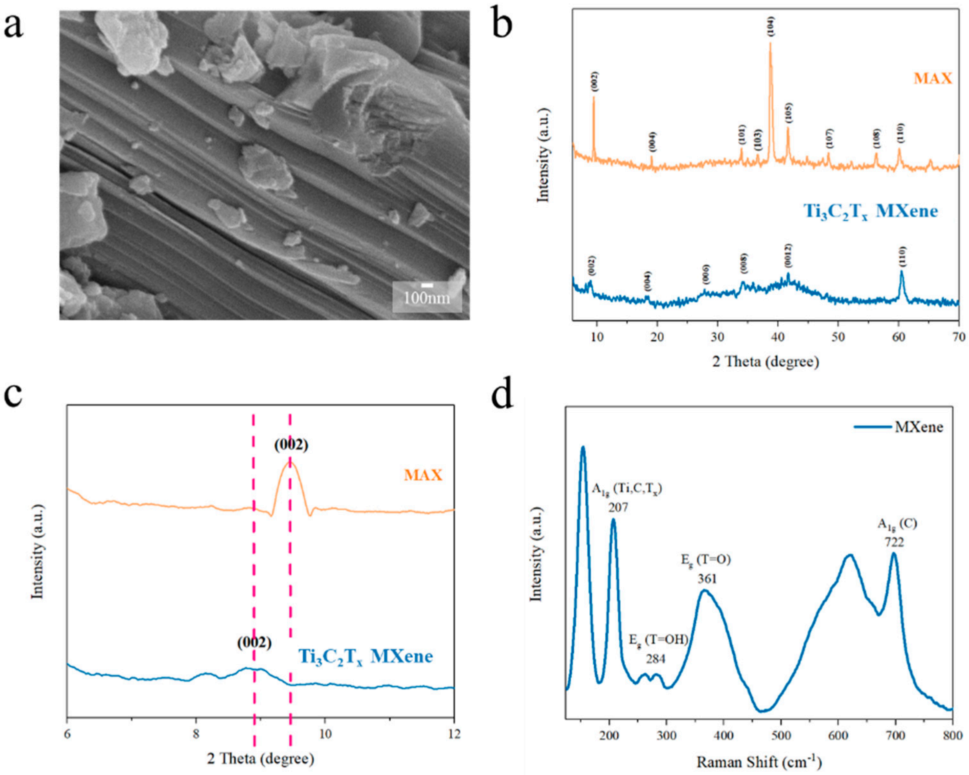


Figure S1. (a) SEM image of MAX. (b,c) XRD patterns of MAX and $Ti_3C_2T_x$ MXene. (d) Raman spectrum of $Ti_3C_2T_x$ MXene.

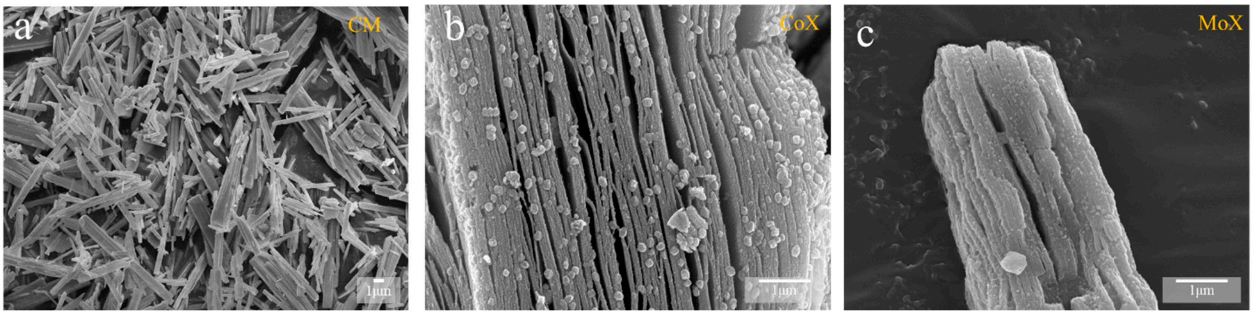


Figure S2. SEM images of (a) CM, (b) CoX, and (c) MoX.

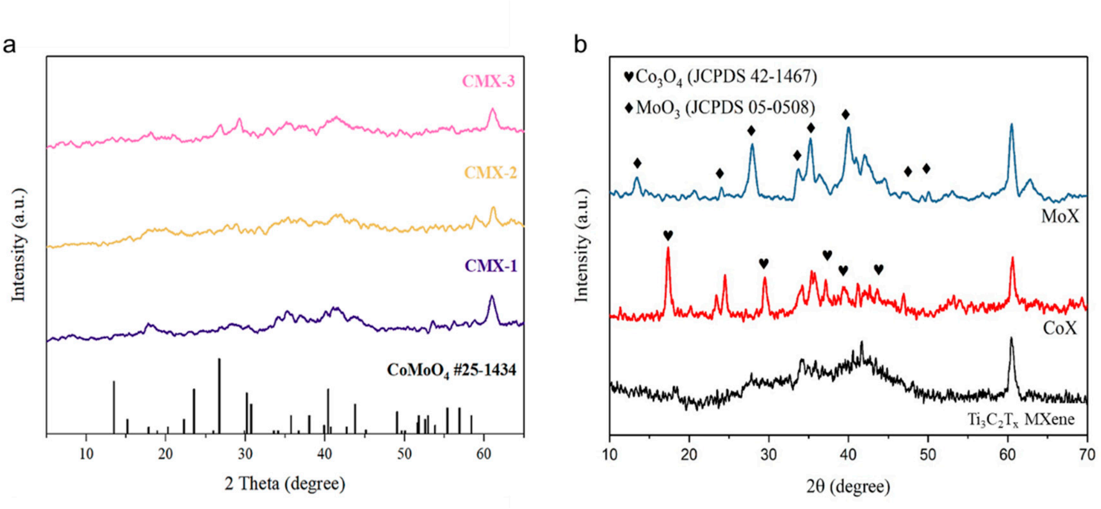


Figure S3. (a) XRD patterns of CMX-1, CMX-2, and CMX-3. (b) XRD patterns of $Ti_3C_2T_x$ MXene, CoX, and MoX.

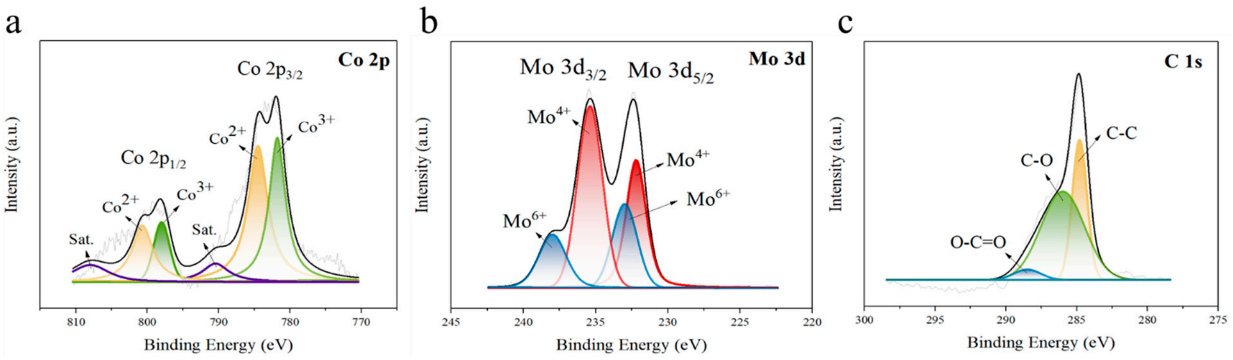


Figure S4. High resolution XPS spectra showing the (a) Co 2p, (b) Mo 3d and (c) C 1s for CMX-1.

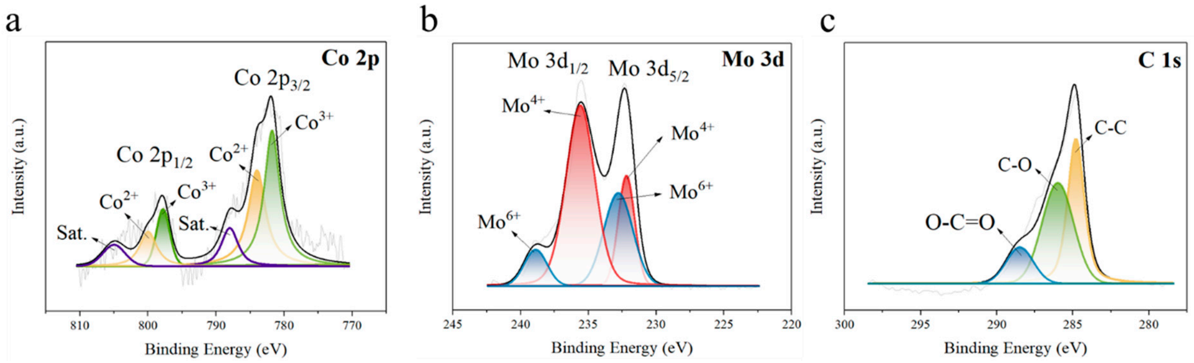


Figure S5. High resolution XPS spectra showing the (a) Co 2p, (b) Mo 3d and (c) C 1s for CMX-3.

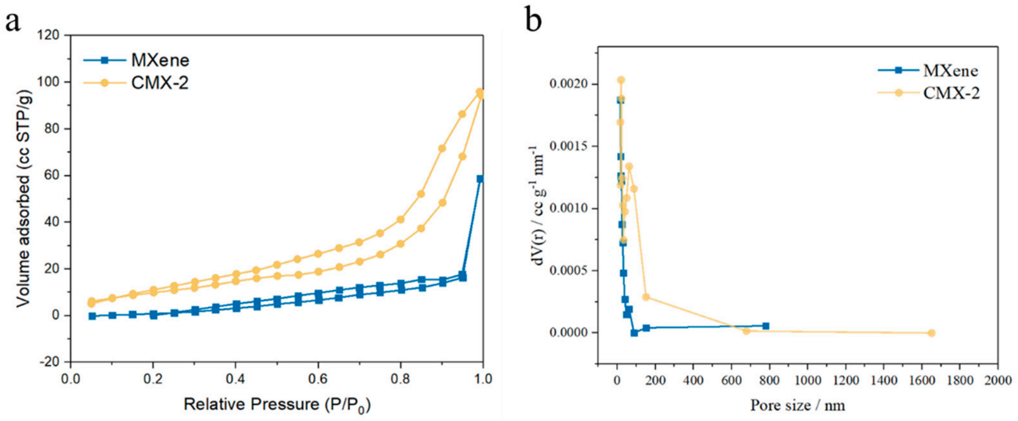


Figure S6. (a) Nitrogen adsorption-desorption isotherms for $Ti_3C_2T_x$ MXene and CMX-2. (b) Pore size distribution of $Ti_3C_2T_x$ MXene and CMX-2 determined using BJH method.

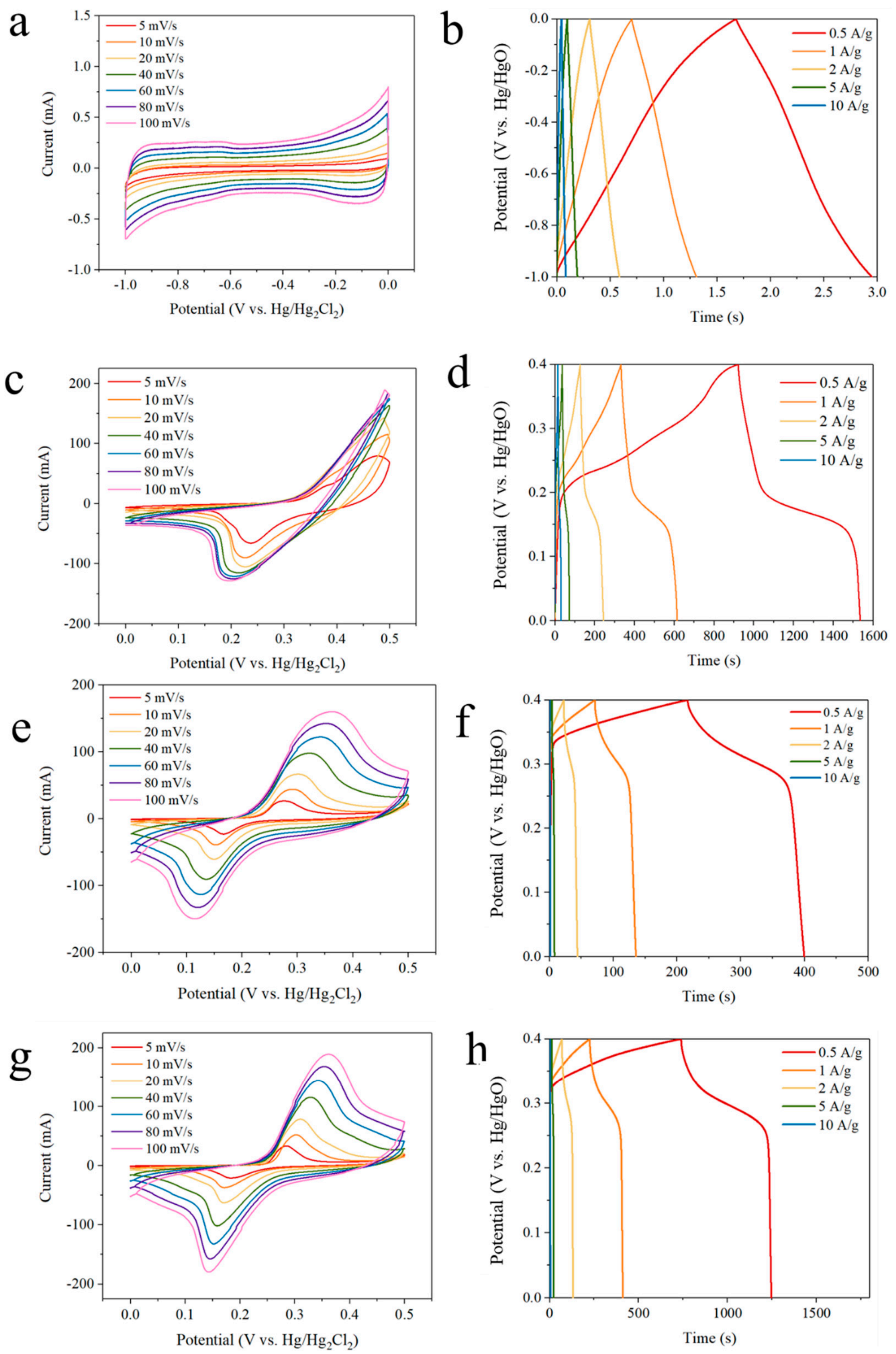

Figure S7. CV curves and GCD curves of (a,b) $Ti_3C_2T_x$ MXene, (c,d) CM, (e,f) CoX, and (g,h) MoX.

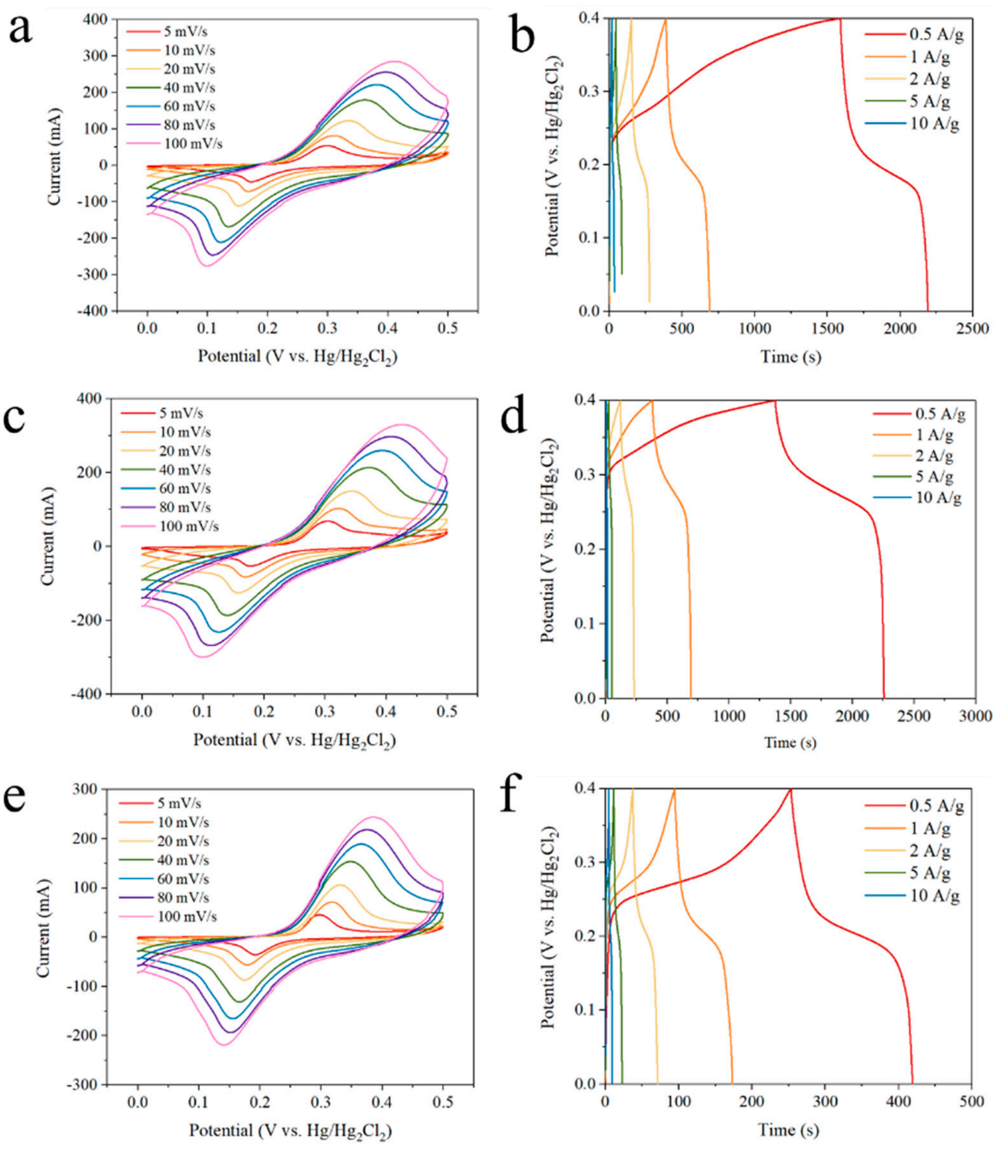


Figure S8. CV curves and GCD curves of (a,b) CMX-1, (c,d) CMX-2, and (e,f) CMX-3.

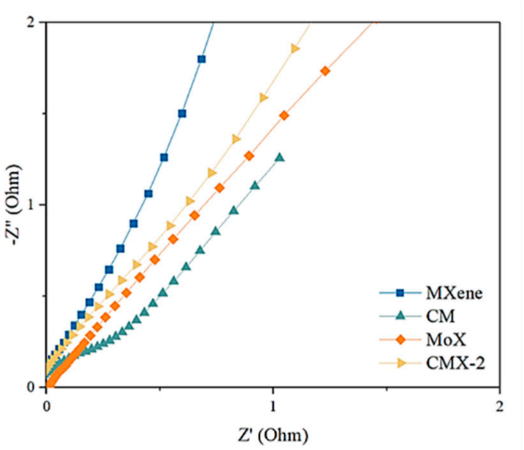


Figure S9. The Nyquist plot of $Ti_3C_2T_x$ MXene, CM, MoX, and CMX-2.

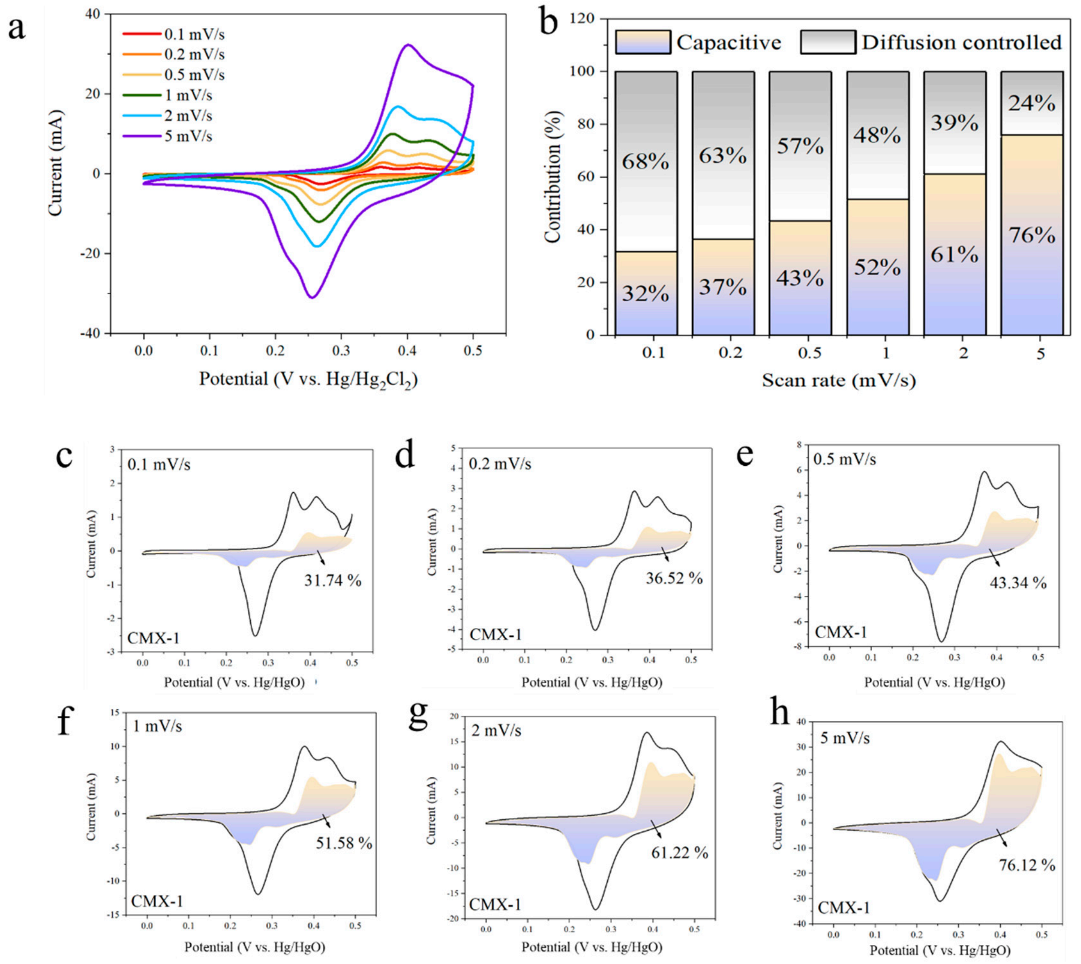


Figure S10. (a) CV curves of CMX-1 at the scan rates of 0.1, 0.2, 0.5, 1, 2, 5 mV $s^{-1}$. (b) Capacitive and diffusion controlled contribution at various scan rates for CMX-1. (c–h) CV curves showing the capacitive and diffusion-controlled contributions at the scan rates ranging from 0.1 to 5 mV $s^{-1}$.

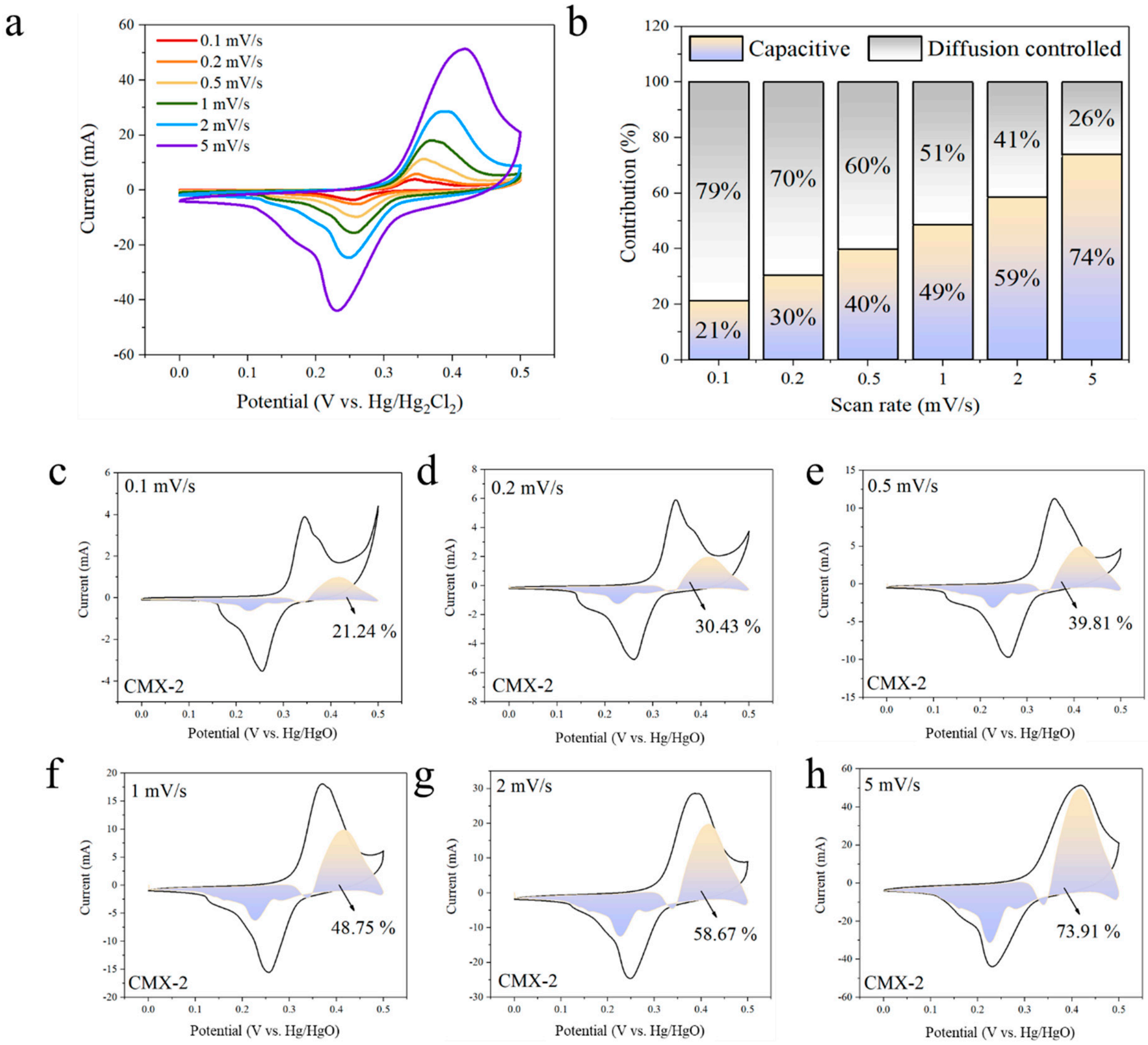


Figure S11. (a) CV curves of CMX-2 at the scan rates of 0.1, 0.2, 0.5, 1, 2, 5 mV $s^{-1}$. (b) Capacitive and diffusion controlled contribution at various scan rates for CMX-2. (c–h) CV curves showing the capacitive and diffusion-controlled contributions at the scan rates ranging from 0.1 to 5 mV $s^{-1}$.

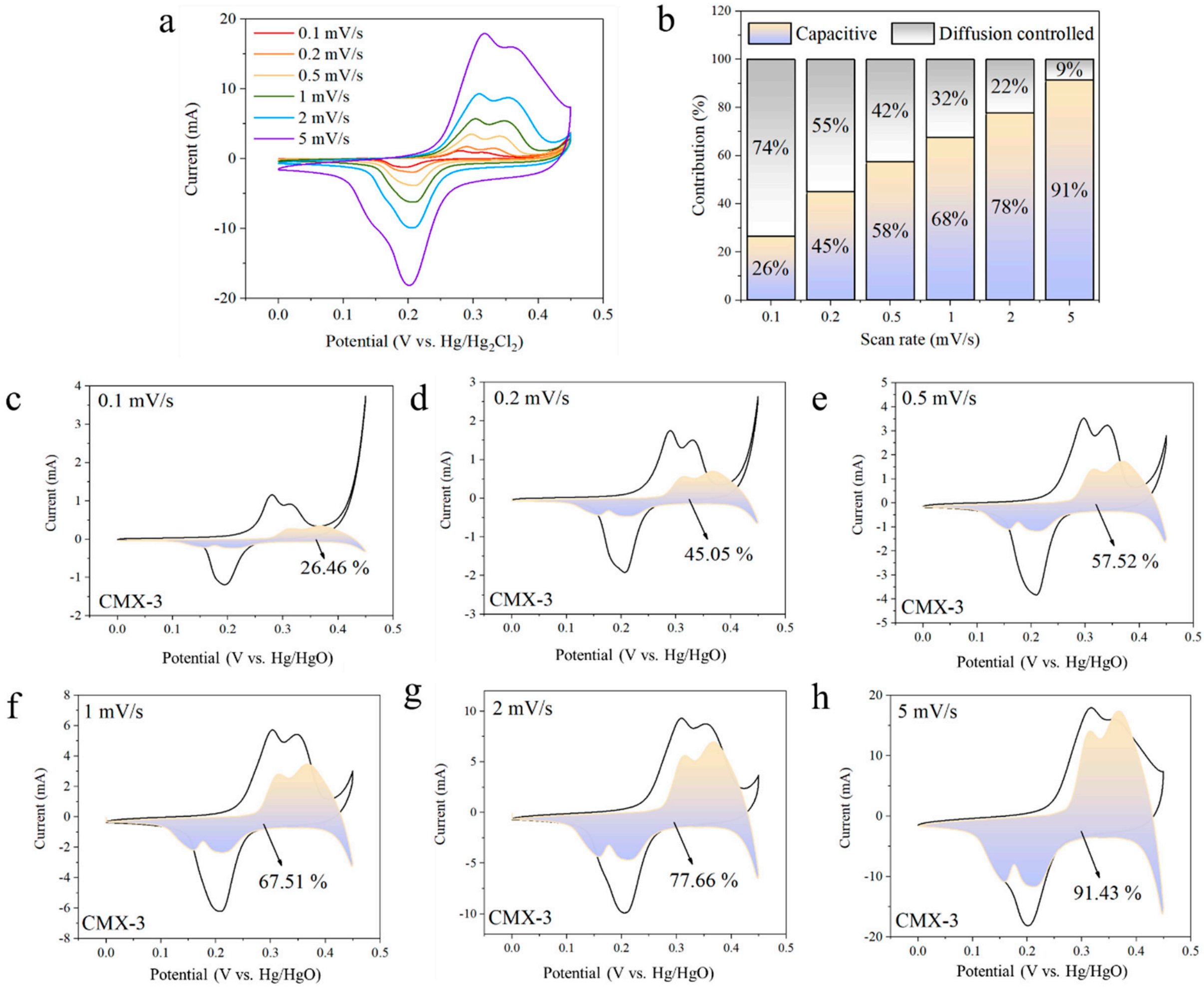


Figure S12. (a) CV curves of CMX-3 at the scan rates of 0.1, 0.2, 0.5, 1, 2, 5 mV $s^{-1}$. (b) Capacitive and diffusion controlled contribution at various scan rates for CMX-3. (c–h) CV curves showing the capacitive and diffusion-controlled contributions at the scan rates ranging from 0.1 to 5 mV $s^{-1}$.

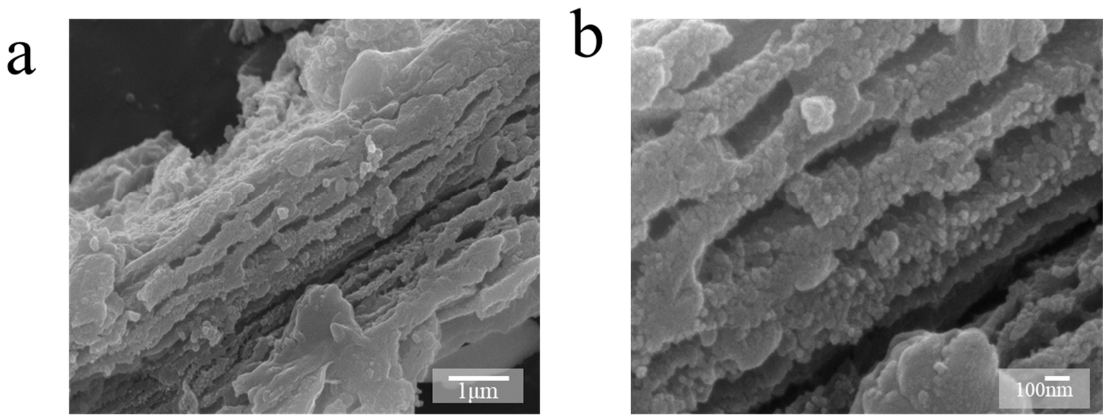


Figure S13. SEM images of CMX-2 after stability test.

Table S1. The element component ratio of CMX-1, CMX-2, and CMX-3.

| Sample | C (at%) | O (at%) | Co (at%) | Mo (at%) | Ti (at%) | Al (at%) |
|---|---|---|---|---|---|---|
| CMX-1 | 50.2 | 31.6 | 10.0 | 6.6 | 1.6 | <0.1 |
| CMX-2 | 60.6 | 28.0 | 5.1 | 4.5 | 1.8 | <0.1 |
| CMX-3 | 58.6 | 27.4 | 4.9 | 6.5 | 2.6 | <0.1 |
